# Psychological Adjustment, Quality of Life and Well-Being in a German and Portuguese Adult Population During COVID-19 Pandemics Crisis

**DOI:** 10.3389/fpsyg.2021.674660

**Published:** 2021-10-15

**Authors:** Adelinda Candeias, Edgar Galindo, Marcus Stueck, António Portelada, Jessica Knietzsch

**Affiliations:** ^1^Comprehensive Health Research Center (CHRC), Universidade de Évora, Évora, Portugal; ^2^DPFA-Academy of Work and Health, Leipzig, Germany; ^3^International Research Academy BIONET, Leipzig, Germany; ^4^Comprehensive Health Research Center (CHRC) and ESE Santarém, Santarém, Portugal

**Keywords:** well-being, quality of life, COVID-19, mental health, optimism, individual differences COVID-19, Mental health

## Abstract

**Introduction:** The pandemics crisis had consequences in psychological adjustment of persons all over the world. The current study analyzes comparatively the topics of quality of life, and well-being, considering as predictors trait anxiety, feeling of threat, difficulty to relax, empathy and pro-social attitude, health care, sleep quality and optimism, in a population of German and Portuguese adults during the pandemics, in order to obtain a deeper understanding of the psychological reactions to crisis across countries and cultures.

**Methods:** A sample of 470 adults divided in three age groups – —young adults (18—34 years), middle-age adults (34–54 years) and old adults (55 years and older)— completed a self-report questionnaire assessing socio-demographic data, quality of life, well-being, quality of sleep, trait anxiety, Coronavirus threat, optimism regarding the pandemics, difficulty to relax, empathy, and pro-social attitude during the pandemics period.

**Results:** Portuguese participants expresses higher empathy and pro-social attitude and health care but in Germany people have higher quality of sleep. Young adults (a) rated their quality of life lower than middle-age adults and old adults, (b) showed also lower optimism than middle-age and old adults, and (c) showed lower well-being than middle-age,.

**Conclusions:** Young adults rated their quality of life, optimism and well-being during pandemics lower than middle-age and old adults, and experienced higher levels of trait anxiety and difficulty to relax. It seems that young adults show a lower psychological adjustment than other age groups during COVID-19 crisis. It is concluded that quality of life, optimism, and well-being during the pandemics are affected differently according to country and group of age, suggesting individual differences across cultures and ages, and consequently the need of specific interventions to cope with the psychological reactions to pandemics crisis.

## Introduction

The COVID-19 pandemics is rocketing around the world. It is universally known that the disease affects several body systems. Notwithstanding, it is becoming more and more evident that also mental health may be heavily affected, not only in infected people, but also in health-care professionals working in hospitals and in the population as a whole. Vindegaard and Benros (2020) searched studies measuring psychiatric symptoms or morbidities associated with COVID-19 among infected patients and among non-infected groups (psychiatric patients, health care workers and non-health care workers). They found a high level of post-traumatic stress and of depressive symptoms in infected persons. On the other hand, compared to before COVID-19, they found a worsening of psychiatric symptoms in patients with preexisting psychiatric disorders, increased depression/depressive symptoms, anxiety, psychological distress, and poor sleep quality in health care workers, and lower psychological well-being and higher scores of anxiety and depression in general public. Negative symptoms were associated to factors like female gender, poor-self-related health and infected relatives.

Patients with COVID-19 not only experience a range of neurological, cognitive and psychiatric symptoms, like dizziness, confusion, seizures and delirium (Fotuhi et al., 2020; Helms et al., 2020; Mukhtar, 2020), but their general well-being can be affected. Stam et al. (2020) describe possible aftereffects of COVID-19 like anxiety disorders, cognitive problems and other difficulties like muscle weakness, severe fatigue and neuropathies.

Non-infected people may present nevertheless some troubles. Beyond fear and contagion danger, the biggest lockdown in the lives of most of the people called dramatic changes in everyday behavior: Wearing masks, physically distancing, avoiding human contacts. The consequences range from PTSD to stress, anxiety, depression, domestic violence, marital problems, and fear before the consequences of financial problems.

Chinese studies conducted during the height of the outbreak in China during 2020, showed serious disruptions of normal life. Zhou et al. (2020) studied 8079 Chinese teens (12 to –18 years old) and found that 44% showed depression, 37% anxiety and 31% both. Li et al. (2020) studied sexual activity of the population in a sample of 459 heterosexual individuals of both genders (8 to –45 years old). They reported a reduction in sexual desire (25%), a decrease in the number of sexual partners (44%), a decrease in frequency of sexual activity (37%) and a reduction in sexual satisfaction (35%). On the other hand, knowledge about COVID-19 seems to lead to more emotional well-being according to Yang and Ma (2020), who conducted a nationwide survey before and after the COVID-19 outbreak. The study found a 74% drop in emotional well-being during the outbreak, but a higher level of self-perceived knowledge about the disease was associated with higher emotional well-being. A special mention was devoted by Chinese researchers to the situation of elderly people, the most vulnerable group in this pandemic. According to Yang et al. (2020), not only the fear of the disease itself, but also the hard limitations to social and familiar life had a negative effect on the emotional and cognitive functioning of the elderly, causing often panic, and anxiety states. These results were corroborated in Spain. González-Sanguino et al. (2020) analyzed the responses of 3,400 persons between 18 and 80 years old to a survey on mental health symptoms and found significant levels of symptoms for depressive disorder (19%), generalized anxiety (19%) and PTSD (16%). The lack of a net of relationships (loneliness) was associated with a higher level of symptoms and women and young people seemed to be more attained than men and elderly. On the other hand, persons who had been infected or had infected relatives were more likely to show these symptoms.

The isolation measures applied in Europe (v.gr. in Portugal and Germany), limited the availability of usual services for vulnerable groups (elderly, persons with chronic or mental diseases) (Armitage and Nellums, 2020; Yao et al., 2020). An additional burden is that the uncertainty about the possibility of becoming ill and dying and about the health of family and friends has heightened dysphoric mental states (Shigemura et al., 2020).

Additionally, it has been found, that alone the fear of COVID-19 can be associated to anxiety disorders and depressive states, in the context of a constant flow of real or fake news on the pandemics (Armitage and Nellums, 2020). Also Garfin et al. (2020) have found that a certain amount of media exposure to the pandemics is associated with psychological distress, like increased anxiety, acute and posttraumatic stress, which in turn amplifies stress responses leading to general health problems like cardiovascular disease, as well as to misguided health protective behaviors.

With the aim of reducing the spread of the virus, the overload of healthcare systems and infection-related mortality, most governments have implemented public health measures (such as lockdown, quarantine, physical, and social distancing) on the population. The impact of these measures can be different. Horesh and Brown (2020) and Tull et al. (2020) found that the effects of lockdown differs from person to person—those who appreciate to be at home may enjoy it, but others may feel frightening the lack of human interaction. For instance, Pirchio et al. (2021) point to the importance of contact with nature, especially in the case of young people, for promoting the well-being, i.e., the lack of outdoor experiences can be a very important in triggering mental health problems. So, COVID-19 is testing the way of life of people all around the world, raising questions on quality of life, and well-being, threat feelings, empathy and pro-social attitude, health care, sleep quality, and optimism, whose answer may be different in different groups of population and in different countries (Bidzan-Bluma et al., 2020).

Current data show that the pandemics is negatively affecting family relations and is limiting seriously the employment of some population sectors, like those working in the service industry. Consequences on mental health may be evident sooner or later (APA, 2020). It is a known fact, that social isolation, especially perceived social isolation increases the risk of cardiovascular, autoimmune, neurocognitive, and mental health problems (Santini et al., 2020). It is known that difficult relationships in the family may undermine the health of the person more than other relationships (Woods et al., 2020). It is also known that losing the own job is detrimental to physical and mental health (Karsten and Moser, 2009). Consequently, increases in mental health concerns have grown (Holmes et al., 2020). It is perhaps too early for a complete understanding of the current COVID-19 pandemics all over the world. It is evident the lack of a theory able to explain the psychological mechanisms and processes that occur in persons and in groups of people in such a situation, especially the effects of restrictions and control measures applied by state agencies. Nevertheless, health psychology, as the study of social, cognitive, and behavioral processes involved in health, illness and healthcare (Johnston, 1994; Ogden, 2012) provides a convenient point of departure for the development of a theoretical framework to understand from a psychological perspective the effects of the current epidemics situation on well-being and quality of life. That health is not only a product of biological processes, but also of beliefs, expectancies, habits, and interpersonal relations is a well-established fact since the end of the twentieth20th century (see Sterling and Eyer, 1981). Since then, a great amount of research has followed. Also, research on topics like burden management and coping strategies in extreme situations, and on stress and burnout (Fink, 2016) are significantly important for such an understanding as Pfefferbaum and North (2020) keenly point out. An important development for the purposes of the current paper is the inclusion in health psychology of the concepts of life satisfaction and quality of life, defining the former as an individual evaluation of the own life, and the latter as the level of general well-being (Bidzan-Bluma et al., 2020), as well as the concept of well-being, defined as the combination of six distinct components of positive psychological functioning (Ryff and Keyes, 1995).

In the context of the current interest to assess the effect of the pandemics, The Academy for Work Health in Leipzig, Germany launched an international research under the name of “Psychological coping, possibilities of crisis intervention and aftercare in companies and institutions for adults, parents and children” for a better understanding of the psychological reactions to coronavirus pandemics crisis in different countries. The present study, included in this major project, analyzes the characterization of perceived quality of life and well-being during the pandemics, on one side, and on the other the relationship to factors like perceived threat of Coronavirus, optimism regarding the pandemics, difficulty to relax, trait anxiety, sleep quality, and empathy and pro-social attitude, in a sample of adult persons from Germany and Portugal.

## Materials and Methods

### Sample

The sample of this study consists of 470 participants from Germany (470 persons) and Portugal (69 persons). In the frame of a series of studies comparing the different countries participating in this project, it was decided to compare in the current study Portugal and Germany, being Germany the country leading the research and Portugal the home of the authors. One more reason was that there were some differences between the two countries in the application of the rules to cope with the pandemic in the same period—April and May 2020. In Portugal, the first two cases were confirmed on 2nd March. On 12th March, the government declared the highest level of emergency. A strict lockdown was immediately applied, people were told to say at home, all the restaurants, bars and night clubs, schools, universities, and social care institutions for elders were completely closed, as well as borders and airports. All work was interrupted and only the professionals of emergency, health, and public security could work physically. All political parties supported the measures and the Portuguese population accepted and accomplished the rules without opposition. These measures were maintained until the end of May 2020, once the first wave had been controlled. Opposition movements (“Negacionistas”) appeared later and remain small.

The first case in Germany was announced on 27th February 2020. A huge outbreak linked to carnival events in North-Rhine-Westphalia appeared during the month of March. On 13th March, the German government closed schools and kindergartens, limited the functioning of the working population and prohibited visits to nursing homes. These measures were sharpened in the following days, always differently in the different German Länder. In some regions, physical contacts were prohibited, in other curfew were imposed, and some borders to some countries were closed. The process of restrictions was applied with different degrees of agreement in the German Länder, with evident opposition of power groups, with critical comments of some political parties and even after some struggle between the federal government and the regional governments. All these measures began to be eliminated after some successes from 15th April on, re-establishing a relative normality by June, in spite of some controlled outbreaks in factories with poor working conditions. Civil opposition movements (“Querdenkers”) appeared from the very beginning.

The mean age was 45.41 (range 16–80, *SD* = 12.72) for German participants and 37.28 (range 18–66, *SD* = 16.21) for Portuguese participants. The sample was divided in three age groups (Table 1):

**TABLE 1:** Participants.

Variables	Age group	Mean	*SD*	N (%)	Labor satisfaction
German	1. Young	28.90	3.72	89 (18.94%)	53 (60.20%)
	2. Middle-age	44.26	6.13	192 (40.85%)	90 (47.40%)
	3. Old	60.62	5.11	115 (24.47%)	65 (56.50%)
Portuguese	1. Young	21.79	3.37	33 (7.02%)	16 (53.30%)
	2. Middle-age	46.50	5.93	22 (4.68%)	12 (53.30%)
	3. Old	59.29	3.79	14 (2.98%)	6 (46.20%)
**Total**		**44.34**	**13.56**	**470 (100%)**	**242 (51.49%)**

1. Young adults: 18–35 years old (*n* = 122).
2. Middle-age adults: 36–54 years old (*n* = 214).
3. Old adults: 55+ (*n* = 129).

The age of German participants (*n* = 401) ranged from 18 to 80 years: Young adults (89) from 18 to 35 years; middle-age adults (192) from 36 to 54 years; and old adults (115) 55 years and older. The age of Portuguese participants (*n* = 69) ranged from 18 to 66 years: Young adults (33) from 18 to 34 years; middle-age adults (22) from 36 to 54 years; and old adults (14) from 55 and older. Concerning labor satisfaction, we could observe that 52.50% from German participants and 49.27% of Portuguese participants are satisfied with their work during the pandemics period. Regarding academic qualifications of German participants, 52.9%, had a graduation degree, 15.7% a bachelor degree, 13.5% a technical education degree, 11.2% a secondary education degree and 4.7% a Ph.D. degree. In Portugal, 46.4% had a graduation degree, 27.5% a secondary education degree, 17.4% a master’s degree, 2.9% a technical education degree and 1.4% a Ph.D. degree.

### Measurement Tools

The main research tool is the Health Cube—Survey—Corona Virus COVID19 (HCSCV-19). The survey comprises questions related to mental health and well-being in several domains of daily life. For the purposes of this study, only a part of the questions have been analyzed, namely, those related to quality of life, trait anxiety, Coronavirus threat, optimism regarding the pandemics, difficulty to relax, life satisfaction, empathy, and pro-social attitude, well-being, and sleep quality during the pandemics period. The Portuguese version has been made by the authors, adapting the original German text to the Portuguese linguistic and cultural context, in order to preserve semantic equivalence in accordance with the ITC Guidelines (Bartram and Hambleton, 2016; Bartram et al., 2018).

The selected part includes:

1. A socio-demographic survey created for this research.
2. Quality of Life was assessed using the mean of 14 items semantic differential scale (also known as a polarity, polarity profile, or impression differential). The sum of the responses was used as a measure of the variable. The short version of the scale was chosen because it measures some features of the long form of the questionnaire more economically. The original version of the semantic differential was developed by Osgood et al. (1957) and is used to assess personality attitudes. Participants are given adjectives to differentiate using bipolar scales. The reliability of the scale in the current study was assessed using Cronbach’s alpha (0.90).

Example of items:

- How do you evaluate your life in the current situation?
1. Frightening ……………………………7. Fearless;
- How do you evaluate your life in the current situation?
1. Insecure ……………………………7. Self-confident.
3. Trait anxiety was measured with the Trait Anxiety Scale (Krohne, 1996), a self-report scale with 10 items (the sum of the responses was used as a measure of the variable). Trait anxiety is a tendency relatively stable at an intraindividual level, but it shows the interindividual propensity to perceive living situations as threatening and to react to them with an augmented state of anxiety. This tool describes how he or she feels at “this very moment” in relation to 26 items presented on a 4-point Likert intensity scale: 1 = “not at all,” 2 = “somewhat,” 3, = “moderately,” 4 = “very much. The reliability of the scale was assessed using Cronbach’s alpha (0.86). Example of items: “I feel frightened”.
4. The valid and reliable instruments of Bidzan-Bluma et al. (2020), have been modified and adapted to develop measures for life satisfaction and Coronavirus threat, optimism regarding the pandemics, difficulty to relax, empathy, and pro-social attitude, and sleep quality during the pandemics period, on a 4-point Likert intensity scale: 1 = “not at all,” 2 = “somewhat,” 3, = “moderately,” 4 = “very much”.” The total score from each one of the scales is calculated by summing the scores obtained in each of the sub-groups of items from each of the measures. Participants were asked to assess the strength of their fears about COVID-19 in relation to:
(a) Coronavirus threat, using a single scale with 3 items, with a good reliability of the scale using Cronbach’s alpha (0.71). Example of item: “Do you experience the situation regarding the Coronavirus as a threat?” (1 = “not at all,” 2 = “somewhat,” 3, = “moderately,” 4 = “very much”).
(b) Optimism regarding the pandemics, using 3 items, with a good reliability of the scale using Cronbach’s alpha (0.81). Example of item: “Are you optimistic regarding a solution?” (1 = “not at all,” 2 = “somewhat,” 3, = “moderately,” 4 = “very much”).
(c) Sleep quality, using 3 items, with a good reliability of the scale using Cronbach’s alpha (0.81). Example of item - —“I had trouble sleeping” (1 = “not at all,” 2 = “somewhat,” 3, = “moderately,” 4 = “very much”).
(d) Difficulty to relax, using 15 items, with a good reliability of the scale using Cronbach’s alpha (0.91). Example of item: “I felt irritable and angry” (1 = “not at all,” 2 = “somewhat,” 3, = “moderately,” 4 = “very much”).
(e) Health care, using 7 items, with a good reliability of the scale using Cronbach’s alpha (0.71). Example of item—“I’m more careful about washing my hands” (1 = “not at all,” 2 = “somewhat,” 3, = “moderately,” 4 = “very much”).
(f) Empathy and pro-social attitude, using 13 items, with a good reliability of the scale using Cronbach’s alpha (0.78). Example of items: “In comparison to before the Coronavirus outbreak, I feel like I’m doing something for society”; In comparison to before the Coronavirus outbreak, I am more concerned about my partnership/family” (1 = “not at all,” 2 = “somewhat,” 3, = “moderately,” 4 = “very much”).
5. General well-being was measured using ten items taken from the Well-being Manifestations Measure Scale (adaptation by Monteiro et al., 2012, of the original EMBEPP by Massé et al., 1998), ranging from none of the time to all of the time, rated on a 5-point Likert-type intensity scale. The total score of psychological well-being is calculated by summing the scores obtained in the ten items, ranging from zero (0) to fifty (50). The reliability of the scale was assessed using Cronbach’s alpha (0.87). Example of items: “I feel happy”.”

### Procedure

As mentioned, this work is part of a larger project entitled: Health Cube—Survey—Corona Virus COVID19, coordinate by DPFA-Academy of Work and Health and reviewed and approved by the Ethical Committee of University of Gdańsk (decision 30/2020).

The present e-survey follows the recommendations for Improving the Quality of Web Surveys, based in the Checklist for Reporting Results of Internet E-Surveys (CHERRIES) (Eysenbach, 2012).

The preparation of the e-survey was preceded by an introduction explaining the identity of the research team, institutional contacts, the purpose of the study, the guarantee of anonymity, confidentiality, and data use only for scientific purposes. After the informed consent were made, the HCSCV-19 has been applied in a single and individual, 45-min online session, by a member of the project team to a group of master degree students (*N* = 10), in order to analyze the usability and functionality of the electronic survey in Google docs.

The final e-survey was disseminated through email and social networks (Facebook, Moodle, WhatsApp, and Instagram and LinkedIn) from 15th April to 30th May in Portugal and Germany. In this tool, an information sheet and a consent form were available on the first page of the questionnaire in both languages. Participants were free to withdraw at any time without giving explanations and no personal identification was requested to guarantee confidentiality. Participants were given no incentives for answering the questionnaire. The system of Google Forms only provides responses for questionnaires with a 100% completion rate. The responses were downloaded as an Excel file and securely stored using a protected database. The present study followed the ethical code for web-based research (Franzke et al., 2019) and conforms to the principles embodied in the Declaration of Helsinki of the World Medical Association (WMA, 2013).

To create the data base, each one of the questionnaires received by the Google docs platform was downloaded and transformed into SPSS Statistics data file (version 24). The data analysis was carried on with software for data processing SPSS (Statistical Package for the Social Sciences).

## Results

The analyses of psychological adjustment, quality of life, well-being, and optimism in German and Portuguese population of adults during COVID-19 pandemics crisis were performed on the basis of descriptive statistics. First, the sample was divided in age groups, based on statistical and theoretical criteria to understand data across the lifespan. Three groups were defined: (1) young adults (18—34 years), (2) middle-age adults (36—54 years), and (3) old adults (55 years and older) (Table 1).

TABThe means, standard deviations, and intercorrelations (Pearson’s r or Spearman’s rho depending on the variable’s scale) were then analyzed for the study variables on the entire sample. Differences between groups (country and age) were examined using an analysis of variance. To finish, with the aim to test the hypothesis regarding the predictors of psychological adjustment, namely, quality of life, well-being, and sleep quality in German and Portuguese population of adults during COVID-19 pandemics crisis, a regression analyses were performed to better understand the predictors of well-being and quality of life during pandemics. Before running the regression analysis, we checked the predictors’ multicollinearity using the Variance Inflation Factor (VIF).

### Descriptive Statistics

The descriptive and correlations analysis (means, standard deviations, and intercorrelations (Pearson’s r or Spearman’s rho depending on the variable scale) for the observed variables on the entire sample are shown in Table 2. It was observed that well-being and quality of life were positively correlated with empathy and pro-social attitude, optimism, sleep quality, Coronavirus threat; on the other hand, age and labour satisfaction were negatively correlated with trait anxiety and difficulty to relax. Sleep quality was positively correlated with quality of life, empathy and pro-social attitude, optimism, Coronavirus threat. And age, level of graduation and labour satisfaction were negatively correlated with trait anxiety, difficulty to relax, heath care and empathy and pro-social attitude during the pandemics period.

**TABLE 2:** Descriptive statistics and intercorrelation matrix for the variables examined in the study.

Variables	*M*	*SD*	1	2	3	4	5	6	7	8	9	10	11	12
1. Age	44.21	13.59	1	–	–	–	–	–	–	–	–	–	–	–
2. Level of education	–	–	0.090	1	–	–	–	–	–	–	–	–	–	–
3. Labor satisfaction	–	–	–0.016	–0.036	1	–	–	–	–	–	–	–	–	–
4. Quality of life	63.35	13.03	0.202**	0.067	0.295**	1	–	–	–	–	–	–	–	–
5. Corona threat	14.69	3.73	0.209**	0.037	0.247**	0.761**	1	–	–	–	–	–	–	–
6. Optimism	9.56	2.81	0.191**	0.047	0.325**	0.748**	0.704**	1	–	–	–	–	–	–
7. Health care	18.90	4.45	–0.076	0.039	0.022	0.047	–0.072	–0.036	1	–	–	–	–	–
8. Pro-social attitude	35.14	5.30	–0.009	0.056	0.032	0.175**	0.061	0.059	0.502**	1	–	–	–	–
9. Difficulty to relax	28.85	8.99	–0.152**	–0.082	–0.204**	–0.329**	–0.438**	–0.450**	0.327**	0.228**	1	–	–	–
10. Sleep quality	10.14	2.24	0.101*	0.069	0.155**	0.271**	0.364**	0.366**	–0.243**	–0.136**	–0.682**	1	–	–
11. Trait anxiety	22.78	8.06	–0.219**	–0.056	–0.249**	–0.438**	–0.541**	–0.516**	0.247**	0.142**	0.691**	–0.575**	1	–
12. Well-being	36.07	6.42	0.119*	0.081	0.112*	0.426**	0.479**	0.319**	0.167**	0.255**	–0.222**	0.177**	–0.364**	1

*Correlation is significant at the 0.05 level (2-tailed).

**Correlation is significant at the 0.01 level (2-tailed).

### Differences Between Groups

To investigate the differences between German and Portuguese groups of participants, an analysis of variance was performed (Table 3). This analysis shows a significant difference among people in Portugal and Germany with respect to anxiety as a trait, difficulty to relax, optimism, well-being, quality of life, health care, and empathy and pro-social attitude during the pandemics confinement. The means and standard deviation scores show that Portuguese participants express greater scores than German in quality of life, well-being, empathy, and pro-social attitude and health care, as well as in anxiety as a trait and difficulty to relax. But German participants express a better sleep quality.

**TABLE 3:** Differences in groups by country.

**Variables**	**Country**		***F***	***p***	**Eta*^2^***
	**Portugal *M* (*SD*)**	**Germany *M* (*SD*)**			
Quality of life	67.69 (11.80)	62.88 (12.80)	7.038	0.008**	0.019
Corona threat	14.86 (2.64)	14.74 (3.79)	0.007	0.932	0.000
Optimism	9.06 (2.70)	9.70 (2.74)	3.201	0.074	0.009
Health care	24.23 (3.12)	17.96 (3.94)	138.226	0.001*	0.281
Pro-social attitude	39.18 (4.13)	34.39 (5.12)	48.096	0.001*	0.120
Difficulty to relax	33.75 (6.96)	27.80 (8.71)	25.131	0.001*	0.066
Sleep quality	9.00 (2.34)	10.37 (2.09)	22.784	0.001*	0.060
Trait anxiety	26.23 (7.93)	21.89 (7.36)	20.816	0.001*	0.056
Well-being	41.28 (5.66)	35.30 (5.97)	53.059	0.001*	0.130

*N = 456; df = 1.*

*p ≤ 0.05, **p ≤ 0.001.

The main differences between countries yielded an effect of size of 28% in the variable health care [*F*(1, 346) = 138.226, *p* = 0.000], of 12% in empathy and pro-social attitude [*F*(1, 346) = 48.096, *p* = 0.000], and of 6% in sleeping quality [*F*(1, 346) = 22.784, *p* = 0.000]. These data indicate a variability in perceived health care, empathic attitude, and sleeping quality in terms of the country. In Portugal people expresses higher empathic attitude and health care but in Germany people have higher quality of sleep.

A factorial Anova was conducted to compare the main effects of country and group of age, as well as, their interaction effects on the examined variables. A factorial Anova was chosen, because the restricted number of participants in one of our samples (Nachtigall et al., 2003) limits the use of other technics as structural analysis models, and with factorial Anova it is possible to observe the main effects of independent variables as well as its interaction, what could provide guidelines for further studies (Marôco, 2014; Tabachnick and Fidell, 2019).

**FIGURE 1:**
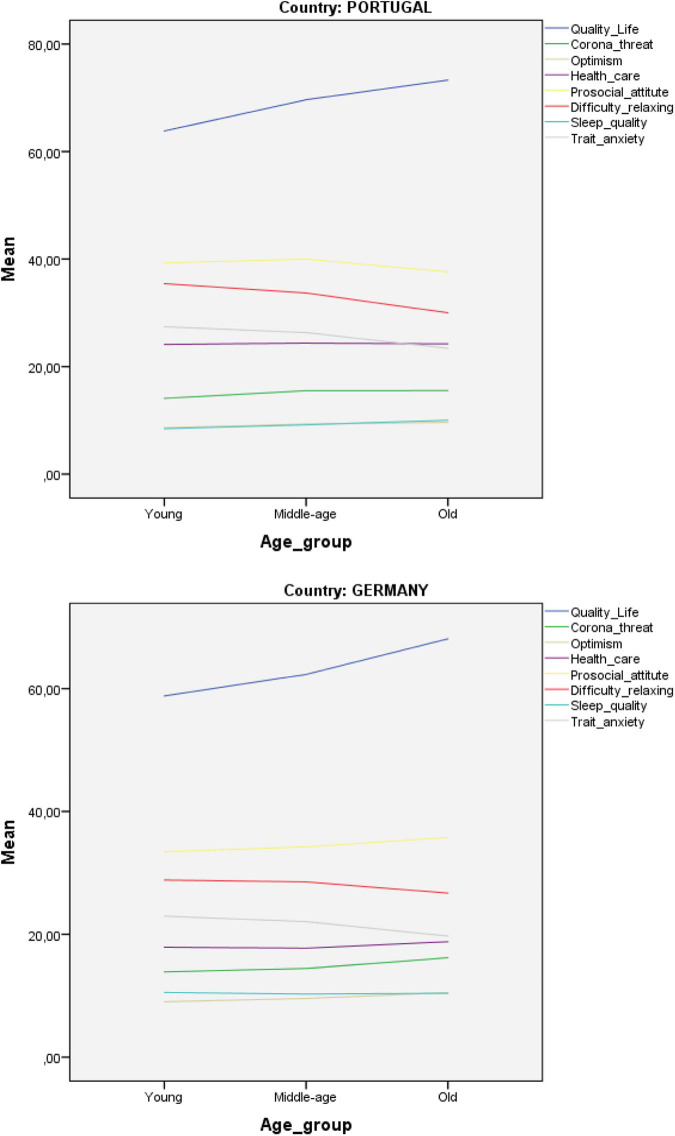
Differences by country and group of age.

Country and group of age effects were statistically significant for the examined variables (see Tables 3, 4, respectively). The multivariate result was significant for country [Roy’s Largest Root = 0.68, *F* = 25.66, df = (1,346), *p* = 0.000], indicating a difference in the variables examined by country. The results were also significant by group of age [Roy’s Largest Root = 0.07, *F* = 2.45, df = (2,346), *p* = 0.000]. Finally, the results were not significant for the interaction between country and group of age [Roy’s Largest Root = 0.04, *F* = 1.48, df = (2,346), *p* = 0.000], indicating that there are no combined effects between country and group of age (see Figure 1).

**TABLE 4:** Differences in group of age.

**Variables**	**Group of age**			***F***	***p***	**Eta^2^**	***Post hoc* Tukey Test**
	**1.Young *M* (*SD*)**	**2. Middle *M* (*SD*)**	**3. Old *M* (*SD*)**				
Quality of life	60.39 (11.70)	63.39 (12.50)	68.81 (11.76)	9.281	0.001**	0.051	G3>G1** G3>G2**
Corona threat	13.94 (3.68)	14.58 (3.89)	16.11 (3.15)	4.071	0.018*	0.023	G3>G1** G3>G2**
Optimism	8.89 (2.71)	9.56 (2.80)	10.40 (2.49)	3.281	0.039*	0.019	G3>G1** G3>G2*
Health care	19.84 (4.59)	18.61 (4.55)	19.54 (4.31)	0.267	0.766	0.002	G1>G2*
Pro-social attitude	35.24 (5.40)	35.07 (5.30)	36.01 (4.80)	0.489	0.614	0.003	–
Difficulty to relax	30.82 (8.80)	29.04 (8.83)	27.16 (8.07)	2.958	0.053	0.017	G1>G2*
Sleep quality	9.89 (2.42)	10.15 (2.14)	10.35 (2.01)	1.703	0.184	0.010	–
Trait anxiety	24.34 (8.22)	22.49 (7.62)	20.21 (6.23)	3.680	0.026*	0.021	G1>G3** G1>G2*
Well-being	35.70 (7.53)	36.22 (6.06)	37.83 (4.96)	3.250	0.040*	0.018	G3>G1*

*N = 456; df = 2.*

*p ≤ 0.05, **p ≤ 0.001.

FIGThe analysis of differences in the examined variables by group of age shows a significant difference among people in different age groups with respect to anxiety as a trait, difficulty to relax, optimism, well-being, and quality of life during the pandemic’s confinement. The means and standard deviation scores based on the Tukey multiple comparisons test show that old adults scored lower than young adults and middle-age adults in anxiety (mean difference = —4.13, SE = —6.60, *p* < 0.01; mean difference = —2.27, SE = —4.51, *p* < 0.01, respectively), and higher than young adults and middle age adults in quality of life (mean difference = 8.42, SE = 1.55, *p* < 0.01; mean difference = 5.42, SE = 1.71, *p* < 0.01, respectively), as well as in Coronavirus threat (mean difference = —2.16, SE = 0.52, *p* < 0.01; mean difference = 1.52, SE = 0.47, *p* < 0.05, respectively). Old adults scored lower than young adults (mean difference = —3.66, SE = 1.20, *p* < 0.05) in difficulty to relax during the pandemics period, and higher than young adults (mean difference = 1.23, SE = 0.31, *p* < 0.01) and middle-aged adults (mean difference = 0.92, SE = 0.30, *p* < 0.05) in optimism during the pandemics period, as well as higher than young adults (mean difference = 2.13, SE = 0.18, *p* < 0.05) in well-being. The main effect of age group was a difference of 5,1% in the variable quality of life [*F*(3, 344) = 9.281, *p* = 0.000], suggesting that 5% of variance in quality of life perceived by participants was explained by age or experience in life.

In the next stage, the predictive power of independent variables was examined, i.e., perceived threat of Coronavirus, optimism regarding the pandemics, difficulty to relax, life satisfaction, health care, empathy, and pro-social attitude and quality of sleep, age, as well as country, and level of education and labour satisfaction relative to dependent variables, namely, well-being and quality of life. A multiple linear regression analysis of variance was performed to understand the impact of the independent variables in dependent variables. First, the residual independence using the Durbin-Watson analysis was examined. The homoscedasticity was investigated, analyzing the plots of residues vs. non-standard predicted values. The absence of multicollinearity was evaluated, taking into account values higher than 0.2. The existence of outliers and tested high scores was analyzed, eliminating studentized residuals greater than ± 3 standard deviations, values greater than.2 and values above 1 for Cook’s distance. Thus, two separate multiple regression analyses were run. For estimating regression coefficients and standard errors, the bootstrap procedure was applied with 1,000 samples. Table 5 shows a summary of the outcomes of these analyses. The analysis of the results obtained demonstrated a significant effect of corona threat in well-being and quality of life [*B* = 29.83, SE = 3.26, β = 0.45, *p* < 0.01; *B* = 27.53, SE = 5.90, β = 0.67, *p* < 0.01, respectively], during the pandemics period. Optimism also appears as a significant and positive predictor of well-being, and anxiety as a trait presents a significant and negative effect. Optimism, health care and age seem to be significant predictors of quality of life.

**TABLE 5:** Summary of results of multiple regression analyses.

Predictors	Well-being		Quality life	
	B [95% CI]	SE	B [95% CI]	SE
Corona threat	29.83** (23.40; 36.16)	3.26	27.53** (16.03; 39.77)	5.90
Optimism	0.66** (0.42; 0.94)	0.13	1.46** (1.10;1.87)	0.20
Health care	–0.04 (–0.35;0.27)	0.16	1.67** (1.16; 2.21)	0.26
Pro-social attitude	0.14 (–0.02; 0.29)	0.08	–0.01 (–0.23; 0.22)	0.12
Difficulty to relax	0.20** (0.08; 0.32)	0.06	0.16 (–0.04; 0.37)	0.10
Sleep quality	0.04 (–0.06; 0.12)	0.05	0.04 (–0.12; 0.16)	0.07
Trait anxiety	–0.26** (–0.37; 0.15)	0.06	0.01 (–0.24; 0.10)	0.09
Age	0.01 (–0.03; 0.04)	0.02	0.07* (0.01; 0.13)	0.03
Country	–5.06** (–6.54; –3.53)	0.76	–5.66** (–8.05; –3.28	1.22
Level of education	0.25 (0.08; 0.61)	0.18	0.10 (–0.55; 0.71)	0.31
Labor satisfaction	–0.57 (–1.67; 0.53)	0.54	1.63 (–0.10; 3.39)	0.85

**Adjusted *R*^2^**	0.45		0.67	

The bold value indicates, **B**, unstandardized regression coefficient; **CI**, confidence interval; **SE**, standard error. N = 352, *p < 0.05, **p < 0.01, bootstrap results are based on 1,000.

## Discussion

Quality of life and well-being during pandemics seem to differ in terms of contextual variables (country) and group of age. In the current investigation, Portuguese people rated well-being, quality of life, health care and empathy and pro-social attitude better than German people, but Portuguese also express higher levels of anxiety as a trait, difficulty to relax, and difficulties to sleep. Germans declare to experience better quality of sleep, lower anxiety, and lower difficulties to relax, but this does not seem to foster a better perception of quality of life and well-being. These data suggest that that the concern of Portuguese adults for others, shown through empathy and pro-social attitudes, gives a sense to confinement in terms of being useful for themselves and for others, also promoting a higher appreciation of health care behavior and a higher perception of positive well-being, a fact that coincides with the results obtained in previous studies (Haramati, 2015; Vinayak and Judge, 2018).

Concerning quality of life and well-being during pandemics, these results seem to indicate, surprisingly, that old adults express higher quality of life, higher optimism, higher well-being, as well as less concern on Corona threat than young adults. Previous studies showed that old people present in general lower levels of quality of life (see for example, Gwozdz and Sousa-Poza, 2010; Huong et al., 2017). It must be mentioned that the current data were collected with a sample, where between 65% (in Germany) and 73% (in Portugal) of participants had high academic qualification and high labor satisfaction (bachelor, graduation, master’s degree, and PhD), a difference in comparison to the study of Bidzan-Bluma et al. (2020). Therefore, it seems that higher levels of quality of life and well-being in old adults are associated with socioeconomic security provided by labor or retirement conditions. On the other hand, these results indicate that young participants show more anxiety as a trait, more difficulty to relax and higher health care (using masks, maintaining social distancing, and washing frequently the hands), suggesting that, during COVID-19 pandemics crisis, young adults show a lower psychological adjustment associated to lower levels of quality of life, well-being, quality of sleep, and optimism than other age groups, a fact that has been observed in other recent studies on the psychological adjustment during pandemic (Lin et al., 2020). One more possible explanation for these results is the fact, found by Pirchio et al. (2021), that the lack of contact with nature seems to affect especially young people.

The results from this study suggest that wellbeing is affected by the concern with the risk of being infected. The concept of perception of the risk (i.e., beliefs, knowledge, values, and attitudes that can influence decisions and behaviors) has become increasingly relevant in research about the current pandemics, as Repišti et al. (2020) refer, creating conditions of psychological vulnerability and mental health risk (Holmes et al., 2020). The current findings are is accordance with other studies that demonstrate a lower quality of life during pandemics, because lockdown produces a sudden change in peoples life, creating a sense of undefinition about the future and the worry about health, as well as limitations in social life (Epifanio et al., 2021). Surprisingly, in the current study age seems to be a predictor of quality of life. One is tempted to believe that a greater experience of life, a higher economic stability and higher labour satisfaction are protective factors of quality of life. Finally, a personality characteristic like optimism seems to be an important predictor of quality of life and well-being and consequently, a predictor of the psychological strength to adjust to all barriers created by Corona pandemics, as Pellerin and Raufaste (2020) also demonstrated with a study conducted in France during the lockdown of April 2020.

Regarding the limitations of this study, the first one is the difference between the samples collected in Germany and in Portugal, the latter being significantly smaller. Secondly, the study was conducted in digital and online format, so that people who did not have access to these means could not participate. On the other hand, given that this is a cross-sectional study, carried out at a certain point in time, it does not allow a comparison between the pre-pandemics period and the period during the pandemics, nor of the effects of the parasite factors (socioeconomic level, health and isolation) in the sample.

Finally, the fact that this is a recent phenomenon makes difficult a comparison with other studies and, in terms of results, it is presently impossible to assess the long-term impact of this pandemics on the various dimensions under study. Future research will be certainly necessary, given the impact of the pandemics. Qualitative and quantitative studies should be done to compare the psychological effects of the pandemics and of each one of the different measures applied by the governments to control the disaster (like social isolation, quarantine, etc.), on different populations and in different countries. It is also important to explore individual differences in coping strategies before the disaster, to develop differential intervention strategies to give psychological support to the affected populations.

## Conclusion

Worldwide, there has been great concern on the effects of the COVID-19 pandemics on the mental health of the population, along with a difficulty in assessing its impact at a more holistic level, due to the individual differences in psychological processes, environment, level of health and SES, leaving to different individual responses to the economic and isolation restrictions applied by the government in every country. This study explores some individual differences concerning the response to the crisis, mainly in terms of the age and nationality, namely, between German and Portuguese adults of three different age groups.

In this study, a higher score in quality of life, optimism and well-being was observed in old adults compared to young and middle-age adults. In the young adults’ group, higher levels of trait anxiety and difficulty to relax were observed in comparison to the other groups.

Regarding the participants’ nationality, Portuguese participants showed higher scores than German participants in quality of life, well-being, empathy and pro-social attitude, and health care, as well as in trait anxiety and difficulty to relax. On the other hand, the German participants expressed a better sleep quality than Portuguese participants. Such results could be interpreted as indicators of profound differences in psychological adjustment to COVID-19 challenges, in different groups of age and in different countries, that should be carefully studied in other countries with more robust samples. Possibly, the measures of social isolation and lockdown applied differently in each country could have impact on psychological adjustment. Portugal is a small country, with central rules for all population, and Germany has applied more varied and flexible rules for different regions. Centralized and well-defined rules seem to correlate with more sense of quality of life, more empathy and more pro-social attitude. These psychological factors could correlate with the fact that centralized measures seem to produce quicker and better results. These possibilities need to be more deeply explored in other countries, with more strong samples.

The present study shows the crucial importance of the protection of mental health during pandemics, in order to moderate the effects of the perceived threat against the personal and social security, especially in the case of young participants, who show a higher psychological vulnerability than other age groups during the COVID-19 pandemics crisis, independently of the country. Finally, quality of life and well-being are influenced negatively by Corona threat, but optimism seems to be a protective factor, suggesting the importance of cultivating a positive perspective in order to cope better with the disaster.

## Data Availability Statement

The raw data supporting the conclusions of this article will be made available by the authors, without undue reservation.

## Ethics Statement

The studies involving human participants were reviewed and approved by the Ethic Committee from DPFA, Germany. The patients/participants provided their written informed consent to participate in this study.

## Author Contributions

AC, EG, and MS: conceptualization, methodology, and supervision. EG and AC: formal analysis. AC, EG, AP, and JK: investigation. EG, AP, and JK: writing–review and editing. All authors contributed to the article and approved the submitted version.

## Conflict of Interest

The authors declare that the research was conducted in the absence of any commercial or financial relationships that could be construed as a potential conflict of interest.

## Publisher’s Note

All claims expressed in this article are solely those of the authors and do not necessarily represent those of their affiliated organizations, or those of the publisher, the editors and the reviewers. Any product that may be evaluated in this article, or claim that may be made by its manufacturer, is not guaranteed or endorsed by the publisher.

## References

[B1] APA (2020). *Work and Unemployment in the Time of COVID-19: Mental Health and Work-Based Implications. Document of APA Division 17 (Counseling Psychology).* Washington, DC: APA.

[B2] Armitage R. Nellums L. B. (2020). COVID-19 and the consequences of isolating the elderly. *Lancet Public Health* 5:e256. 10.1016/S2468-2667(20)30061-X PMC7104160 32199471

[B3] Bartram D. Berberogl G. Grégoire J. Van de Vijver F. (2018). ITC guidelines for translating and adapting tests (second edition). *Int. J. Test.* 18 101–134. 10.1080/15305058.2017.1398166

[B4] Bartram D. Hambleton R. K. (2016). “The ITC guidelines: international standards and guidelines relating to tests and testing,” in *The ITC International Handbook of Testing and Assessment*, eds Leong F. T. L. Bartram D. Cheung F. M. Geisinger K. F. Iliescu D. (Oxford University Press), 35–46. 10.1093/med:psych/9780199356942.003.0004

[B5] Bidzan-Bluma I. Bidzan M. Jurek P. Bidzan L. Knietzsch J. Stueck M. (2020). A polish and German population study of quality of life, well-being, and life satisfaction in older adults during the COVID-19 pandemic. *Front. Psychiatry* 11:585813. 10.3389/fpsyt.2020.585813 33281646 PMC7705096

[B6] Epifanio M. S. Andrei F. Mancini G. Agostini F. Piombo M. A. Spicuzza V. (2021). The impact of COVID-19 pandemic and lockdown measures on quality of life among Italian general population. *J. Clin. Med.* 10:289. 10.3390/jcm10020289 33466778 PMC7830623

[B7] Eysenbach G. (2012). Correction: improving the quality of web surveys: the checklist for reporting results of internet E-surveys (CHERRIES). *J. Med. Internet Res.* 14:e8. 10.2196/jmir.2042 PMC1550605 15471760

[B8] Fink G. (2016). *Stress: Concepts, Cognition, Emotion, and Behavior.* London: Elsevier.

[B9] Fotuhi M. Mian A. Meysami S. Raji C. A. (2020). Neurobiology of COVID-19. *J. Alzheimer Dis.* 76 3–19. 10.3233/jad-200581 32538857 PMC7660990

[B10] Franzke A. Bechmann A. Zimmer M. Ess C. (2019). *Internet Research: Ethical Guidelines 3.0. Association of Internet Researchers.* Available online at: https://aoir.org/reports/ethics3.pdf (accessed April 19).

[B11] Garfin D. R. Silver R. C. Holman E. A. (2020). The novel coronavirus (COVID-2029) outbreak: amplification of public health consequences by media exposure. *Health Psychol.* 39 355–357. 10.1037/hea0000875 32202824 PMC7735659

[B12] González-Sanguino C. Ausín B. Castellanos M. Á Saiz J. López-Gómez A. Ugidos C. (2020). Mental health consequences during the initial stage of the 2020 Coronavirus pandemics (COVID-19) in Spain. *Brain Behav. Immun.* 87 172–176. 10.1016/bbi.2020.05.040 32405150 PMC7219372

[B13] Gwozdz W. Sousa-Poza A. (2010). Ageing, health and life satisfaction of the oldest old: an analysis for Germany. *Soc. Indic. Res.* 97 397–417. 10.1007/s11205-009-9508-8

[B14] Haramati A. (2015). Resilience, empathy and well-being in the health professions: an educational imperative. *Global Adv. Health Med.* 4 5–6. 10.7453/gahmj.2015.092 26421226 PMC4563897

[B15] Helms J. Kremer S. Merdji H. Clere-Jehl R. Schenck M. Kummerlen C. (2020). Neurologic features in severe SARS-CoV-2 infection. *N. Engl. J. Med.* 382 2268–2270. 10.1056/NEJMc2008597 32294339 PMC7179967

[B16] Holmes E. A. O’Connor R. C. Perry V. H. (2020). Multidisciplinary research priorities for the COVID-19 pandemic: a call for action for mental health science. *Lancet Psychiatry* 7 547–560. 32304649 10.1016/S2215-0366(20)30168-1 PMC7159850

[B17] Horesh D. Brown A. D. (2020). Traumatic stress in the age of COVID-19: a call to close critical gaps and adapt to new realities. *Psychol. Trauma* 12 331–335. 10.1037/tra0000592 32271070

[B18] Huong N. T. Ha L. T. H. Tiene T. Q. (2017). Determinants of health-related quality of life among elderly: evidence from Chi Linh town, Vietnam. *Asia Pac. J. Public Health* 29 84–93. 10.1177/1010539517704041 28425322

[B19] Johnston M. (1994). Current trends in health psychology. *Psychologist* 7 114–118.

[B20] Karsten P. Moser M. (2009). Unemployment impairs mental health: meta-analyses. *J. Vocat. Behav.* 74 264–282. 10.1016/j.jvb.2009.01.001

[B21] Krohne H. W. (1996). *Anxiety und Coping.* Stuttgart: Kohlhammer.

[B22] Li W. Li G. Xin C. Wang Y. Yang S. (2020). Challenges in the practice of sexual medicine in the time of COVID-19 in China. *J. Sex. Med.* 17 1225–1228. 10.1016/j.jsxm.2020.04.380 32418751 PMC7188657

[B23] Lin Y. Hu Z. Alias H. Wong L. P. (2020). Knowledge, attitudes, impact, and anxiety regarding COVID-19 infection among the public in china. *Front. Public Health* 8:236. 10.3389/fpubh.2020.00236 32574305 PMC7266871

[B24] Marôco J. P. (2014). *Análise Estatística Com o SPSS Statistics*, 6th Edn. Pinheiro:: Livraria Almedina.

[B25] Massé R. Poulin C. Dassa C. Lambert J. Bélair S. Battaglini A. (1998). Élaboration et validation d’un outil de mesure du bien-être psychologique: L’É.M.M.B.E.P. (Elaboration and validation of instrument to measure the psychological well.being: the EMMBEP). *Can. J. Public Health* 89 352–357. 10.1007/BF03404490 9813928 PMC6990188

[B26] Monteiro S. Tavares J. Pereira A. (2012). Adaptação portuguesa da escala de medida de manifestação de bem-estar psicológico com estudantes universitários (Portugese adaptation of a scale to measure psychological well.being in university students). *Psicologia Saúde Doenças* 13 66–77.

[B27] Mukhtar S. (2020). Psychological health during the coronavirus disease 2019 pandemics outbreak. *Int. J. Soc. Psychiatry* 66 512–516. 10.1177/0020764020925835 32434402 PMC7405632

[B28] Nachtigall C. Kroehne U. Funke F. Steyer R. (2003). (Why) should we use SEM? Pros and cons of structural equation modeling. *Methods Psychol. Res.* 8 1–22. 10.1080/10705511.2021.1935263

[B29] Ogden J. (2012). *Health Psychology: A Textbook*, 5th Edn. Maidenhead: Open University Press.

[B30] Osgood C. E. Suci G. J. Tannenbaum P. H. (1957). *The Measurement of Meaning.* Champaign, IL: University of Illinois Press.

[B31] Pellerin N. Raufaste E. (2020). Psychological resources protect well-being during the covid-19 pandemic: a longitudinal study during the french lockdown. *Front. Psychol*. 11:590276. 10.3389/fpsyg.2020.590276 33424709 PMC7793808

[B32] Pfefferbaum B. North C. S. (2020). Mental health and the Covid-19 pandemics. *N. Eng. J. Med.* 383 510–512. 10.1056/NEJMp2008017 32283003

[B33] Pirchio S. Passiatore Y. Panno A. Cipparone M. Carrus G. (2021). The effects of contact with nature during outdoor environmental education on students’ wellbeing, connectedness to nature and pro-sociality. *Front. Psychol.* 12:1523. 10.3389/fpsyg.2021.648458 34017288 PMC8129515

[B34] Repišti S. Jovanoviæ N. Kuzman M. R. Medved S. Jerotiæ S. Ribiæ E. (2020). How to measure the impact of the COVID-19 pandemic on quality of life: COV19-QoL–the development, reliability and validity of a new scale. *Global Psychiatry* 3 201–210. 10.2478/gp-2020-0016

[B35] Ryff C. D. Keyes C. M. (1995). The structure of psychological well-being revisited. *J. Pers. Soc. Psychol.* 69 719–727. 10.1037/0022-3514.69.4.719 7473027

[B36] Santini Z. Jose P. Cornwell E. Koyanagi A. Nielsen L. Hinrichsen C. (2020). Social disconnectedness, perceived isolation, and symptoms of depression and anxiety among older Americans (NSHAP): a longitudinal mediation analysis. *Lancet Public Health* 5 62–70. 10.1016/S2468-2667(19)30230-0 31910981

[B37] Shigemura J. Ursano R. J. Morganstein J. C. Kurosawa M. Benedek D. M. (2020). Public responses to the novel 2019 coronavirus (2019-nCoV) in Japan: mental health consequences and target populations. *Psychiatry Clin. Neurosci.* 74 281–282. 10.1111/pcn.12988 32034840 PMC7168047

[B38] Stam H. J. Stucki G. Bickenbach J. (2020). COVID-19 and post intensive care syndrome: a call for action. *J. Rehabil. Med.* 52:jrm00044. 10.2340/16501977-2677 32286675

[B39] Sterling P. Eyer J. (1981). Biological basis of stress-related mortality. *Soc. Sci. Med. E* 15 3–42. 10.1016/0271-5384(81)90061-2 7020084

[B40] Tabachnick B. G. Fidell L. S. (2019). *Using Multivariate Statistics*, 7th Edn. New York, NY: Harper Collins College Publishers.

[B41] Tull M. T. Edmonds K. A. Scamaldo K. M. Richmond J. R. Rose J. P. Gratz K. L. (2020). Psychological outcomes associated with stay-at-home orders and the perceived impact of COVID-19 on daily life. *Psychiatry Res.* 289:113098. 10.1016/j.psychres.2020.113098 32434092 PMC7252159

[B42] Vinayak S. Judge J. (2018). Resilience and empathy as predictors of psychological well-being among adolescents. *Int. J. Health Sci. Res.* 8 192–200.

[B43] Vindegaard N. Benros M. E. (2020). COVID-19 pandemics and mental health consequences: systematic review of the current evidence. *Brain Behav. Immun.* 89 531–542. 10.1016/j.bbi.2020.05.048 32485289 PMC7260522

[B44] Woods S. B. Priest J. B. Roberson P. N. E. (2020). Family versus intimate partners: estimating who matters more for health in a 20-year longitudinal study. *J. Fam. Psychol.* 34 247–256. 10.1037/fam0000600 31697103 PMC7012715

[B45] WMA (2013). World Medical Association declaration of helsinki: ethical principles for medical research involving human subjects. *JAMA* 310 2191–2194. 10.1001/jama.2013.281053 24141714

[B46] Yang H. Ma J. (2020). How an epidemic outbreak impacts happiness: factors that worsen (vs. Protect) emotional well-being during the coronavirus pandemics. *Psychiatry Res.* 289:113045. 10.1016/j.psychres.2020.113045 PMC7190485 32388418

[B47] Yang Y. Li W. Zhang Q. Zhang L. Cheung T. Xiang Y. T. (2020). Mental health services for older adults in China during the COVID-19 outbreak. *Lancet Psychiatry* 7:19. 10.1016/S2215-0366(20)30079-1 PMC7128970 32085843

[B48] Yao H. Chen J. H. Xu Y. F. (2020). Patients with mental health disorders in the COVID-19 epidemic. *Lancet Psychiatry* 7:21. 10.1016/S2215-0366(20)30090-0 PMC7269717 32199510

[B49] Zhou S.-J. Zhang L. G. Wang L. L. Guo Z. C. Wang J. Q. Chen J. C. (2020). Prevalence and socio-demographic correlates of psychological health problems in Chinese adolescents during the outbreak of COVID-19. *Eur. Child Adolesc. Psychiatry* 29 749–758. 10.1007/s00787-020-01541-4 32363492 PMC7196181

